# *Yarrowia lipolytica* growth, lipids, and protease production in medium with higher alkanes and alkenes

**DOI:** 10.1007/s11274-024-04123-7

**Published:** 2024-09-12

**Authors:** Sílvia M. Miranda, Isabel Belo, Marlene Lopes

**Affiliations:** 1 Centre of Biological Engineering, University of Minho, Campus de Gualtar, 4710-057 Braga, Portugal; 2 LABBELS – Associate Laboratory, Braga/Guimarães, Portugal

**Keywords:** Aliphatic hydrocarbons, Extracellular enzymes, Lipids production, Oxygen supply, *Yarrowia lipolytica*

## Abstract

Two strains of *Yarrowia lipolytica* (CBS 2075 and DSM 8218) were first studied in bioreactor batch cultures, under different controlled dissolved oxygen concentrations (DOC), to assess their ability to assimilate aliphatic hydrocarbons (HC) as a carbon source in a mixture containing 2 g·L^−1^ of each alkane (dodecane and hexadecane), and 2 g·L^−1^ hexadecene. Both strains grew in the HC mixture without a lag phase, and for both strains, 30 % DOC was sufficient to reach the maximum values of biomass and lipids. To enhance lipid-rich biomass and enzyme production, a pulse fed-batch strategy was tested, for the first time, with the addition of one or three pulses of concentrated HC medium. The addition of three pulses of the HC mixture (total of 24 g·L^−1^ HC) did not hinder cell proliferation, and high protease (> 3000 U·L^−1^) and lipids concentrations of 3.4 g·L^−1^ and 4.3 g·L^−1^ were achieved in *Y. lipolytica* CBS 2075 and DSM 8218 cultures, respectively. Lipids from the CBS 2075 strain are rich in C16:0 and C18:1, resembling the composition of palm oil, considered suitable for the biodiesel industry. Lipids from the DSM 8218 strain were predominantly composed of C16:0 and C16:1, the latter being a valuable monounsaturated fatty acid used in the pharmaceutical industry. *Y. lipolytica* cells exhibited high intrinsic surface hydrophobicity (> 69 %), which increased in the presence of HC. A reduction in surface tension was observed in both *Y. lipolytica* cultures, suggesting the production of extracellular biosurfactants, even at low amounts. This study marks a significant advancement in the valorization of HC for producing high-value products by exploring the hydrophobic compounds metabolism of *Y. lipolytica*.

## Introduction

Aliphatic hydrocarbons (HC) (e.g., alkanes and alkenes) have diverse applications in industry and are used as raw material for the synthesis of various chemicals, liquid transportation fuels (e.g., gasoline, diesel, and jet fuel), lubricants, and plastics (Kuppusamy et al. 2020). As one of the higher *n*-alkanes, *n*-dodecane is often employed as the surrogate or a surrogate component for kerosene, jet fuel, and diesel (Mao et al. 2020). Hexadecane is one of the major components of diesel fuel, ranging from a few percent to over 10 %, depending on factors such as the refinery process used and additives included in the fuel formulation (Krummenacher et al. 2003). This aliphatic hydrocarbon is also one of the predominant alkanes present in hydrotreated vegetable oil, which is used as a biofuel (Tang et al. 2018). Furthermore, hexadecane is used as a base material in the production of most commercial organic lubricants (Cafolla and Voïtchovsky 2020). Hexadecene, a linear alpha-olefin, is widely used as a raw material for chemical products such as synthetic plasticizers, synthetic detergents, and lubricants (Yang et al. 2024).

Due to the broad use of the above-mentioned HC in high volumes in many products and formulations is inevitable that they are transferred to wastewater treatment plants or soil. Considering the important role of aliphatic hydrocarbons in chemical synthesis and industrial production and the environmental impact owing to their prevalence in terrestrial and aquatic ecosystems, its research has garnered increasing attention over the last decades. To minimize or eradicate environmental pollution by HC contaminants, several physical, chemical, and biological approaches have been studied. Among those strategies, biological treatments are considered one of the most promising eco-friendly options for highly HC-polluted effluents. Microorganisms use these compounds as carbon sources and, in some cases, produce added-value metabolites while reducing organic load and environmental impact (Thorat and Sonwani 2022). However, most relevant studies are focused on the bacteria species (e.g., *Acinetobacter* sp., *Bacillus* sp., *Pseudomonas* sp., *Sphingomonas* sp., *Rhodococcus* sp., and *Alcaligenes* sp.) owing to their ability to utilize such compounds as energy sources and their potential to bioremediate HC-polluted streams (Imron et al. 2020; Ławniczak et al. 2020; Kebede et al. 2021).

*Yarrowia lipolytica* is commonly found in oil-contaminated environments and demonstrates a notable affinity for hydrophobic substrates, including aliphatic HC (Lopes et al. 2022; Miranda et al. 2024) and aromatic compounds (Ferreira et al. 2023). *Y. lipolytica* developed strategies to increase the bioavailability of HC, including the production of extracellular biosurfactants (Ferreira et al. 2023). Additionally, yeast cells can directly adhere to HC droplets due to their hydrophobic cell surfaces (Amaral et al. 2006). Upon entering the cells, alkanes are metabolized via the β-oxidation pathway into acetyl-CoA, which can be incorporated in the glyoxylate, Krebs, or methyl-citrate cycles (Fukuda 2023).

*Yarrowia lipolytica* has garnered considerable scientific interest as a noteworthy cell factory for the secretion of valuable compounds, namely enzymes (Janek et al. 2020), organic acids (Tomaszewska-Hetman et al. 2021), and polyols (Tomaszewska et al. 2014; Rywińska et al. 2024). Additionally, it has demonstrated excellence as a producer of lipids (Papanikolaou and Aggelis 2010; Lazar et al. 2014; Rakicka et al. 2015; Dobrowolski et al. 2019), and recent work (Miranda et al. 2024) proved the ability of the CBS 2075 strain to produce lipid-rich biomass and protease from hexadecane. *Y. lipolytica* DSM 8218, formerly known as *C. lipolytica*, was isolated from fuel storage tanks and possesses a specific ability to metabolize aromatic hydrocarbons such as naphthalene, biphenyl, and benzo(a)pyrene (Cerniglia and Crow 1981). This study intended to test both *Y. lipolytica* strains in a mixture of higher alkanes (dodecane and hexadecane) and alkenes (hexadecene) since no papers have been published so far concerning their application to produce lipids and enzymes from such HC mixture. Firstly, batch cultures were carried out in a lab-scale stirred-tank bioreactor under different controlled dissolved oxygen concentrations (DOC) (30 – 90%) to select the best oxygenation conditions for HC assimilation and biomass, lipids, and enzymes (protease and lipase) production. As a prospect to improve lipid-rich biomass and extracellular enzyme production by both *Y. lipolytica* strains, a pulse fed-batch approach was attempted at 30 % DOC. In this strategy, intermittent feeding of concentrated HC medium (by one or three pulses) after HC depletion was applied as a strategy to supply more substrate to the yeast cells preventing the potential inhibitory effect of high initial HC concentration.

## Materials and methods

### Yeast strains preservation and inoculum preparation

*Yarrowia lipolytica* CBS 2075 and *Y. lipolytica* DSM 8218 grew overnight in YPD medium (20 g·L^−1^ glucose, 20 g·L^−1^ peptone, and 10 g·L^−1^ yeast extract) to prepare cryo-stocks in sterile microtubes (800 μL of yeast culture and 200 μL of pure glycerol), which were preserved at − 80 °C. Pre-inoculum cultures (150 mL of YPD medium) were inoculated with a thawed culture of one microtube and placed in an orbital incubator at 27 °C and 200 rpm. After 16 h of growth, yeast cells were harvested by centrifugation and resuspended in the culture medium at an initial dry cell concentration of 0.5 g·L^−1^.

### Batch cultures

Batch cultures were carried out in 2-L stirred tank bioreactors (DASGIP Parallel Bioreactor System, Eppendorf, Germany) with 1.2-L of medium composed of 6 g·L^−1^ HC mixture (2 g·L^−1^ dodecane, 2 g·L^−1^ hexadecane, and 2 g·L^−1^ hexadecene), 0.5 g·L^−1^ ammonium sulfate, and 3.4 g·L^−1^ corn steep liquor (CSL, C4648-500G, Sigma-Aldrich®) — initial C/N ratio of 48. The experiments were conducted at 27 °C and pH of 5.5 ± 0.5 automatically controlled by the addition of NaOH 2 M or HCl 2 M. To select the best controlled DOC conditions for biomass, lipids and enzyme production by *Y. lipolytica* strains, four conditions of controlled DOC (30 %, 50 %, 70 %, and 90 % of saturation) were studied through a cascade control mode, in which the stirring rate automatically varied between 200 rpm and 800 rpm. The specific airflow rate was set at 3 vvm (90 % DOC), 2 vvm (70 % DOC), and 1 vvm (50 % and 30 % DOC). DOC was monitored with a polarographic oxygen probe (Inpro6820/12/320-type, Mettler Toledo).

### Pulse fed-batch cultures

*Yarrowia lipolytica* strains grew for 48 h in a medium comprising 6 g·L^−1^ of an HC mixture (2 g·L^−1^ dodecane, 2 g·L^−1^ hexadecane and 2 g·L^−1^ hexadecene), 0.5 g·L^−1^ ammonium sulfate, and 3.4 g·L^−1^ CSL (batch stage) — initial C/N ratio of 48, followed by the addition of a single pulse of concentrated medium to attain the initial concentration of each compound. Additional experiments were performed in which batch phase (24 h) was followed by adding three pulses of concentrated medium at 24 h, 48 h, and 72 h. All experiments were carried out at a specific airflow rate of 1 vvm and controlled DOC of 30 % by automatic variation of stirring rate between 200 rpm and 500 rpm.

### Analytical methods

Samples were collected at specific intervals to quantify biomass, HC concentration, extracellular enzyme activity, cell hydrophobicity, and surface tension of the medium. Yeast cells were harvested by centrifugation and stored at − 20 °C for further quantification of intracellular lipids and long-chain fatty acids composition.

Biomass concentration was quantified by the optical density of cultures at 600 nm and converted to cell dry weight (g·L^−1^) using a calibration curve for each yeast strain. Extracellular lipase activity was quantified in samples supernatant according to Miranda et al. (2024), using *p*-nitrophenyl butyrate 1 mM as substrate dissolved in 250 μL of a mixture with phosphate buffer 50 mM (pH 7.3), acetone 4 % (v/v), and Triton-X 4 % (v/v), and following the enzymatic reaction for 10 min at 37 °C. Lipase activity was calculated by linear regression of absorbance *versus* time, using the molar extinction coefficient of *p*-nitrophenol (2.3 mM^−1^). One unit of activity was expressed as the amount of lipase that produces 1 μmol of *p*-nitrophenol per minute under the conditions used. Extracellular protease activity was measured in the culture supernatant using 0.5 % (w/v) azocasein dissolved in sodium acetate buffer 50 mM (pH 5) as substrate at 37 °C for 40 min. One unit of enzyme activity was defined as the amount of enzyme that causes an increase of 0.01 absorbance comparatively to the blank (supernatant was replaced by sodium acetate buffer 50 mM) per minute under assay conditions (Miranda et al. 2024).

The hydrophobicity of the cells’ surface was evaluated by the microbial adhesion to hydrocarbons (MATH) test (Rosenberg et al. 1980). Yeast cells were harvested, centrifuged (8000 rpm, 10 min), washed with phosphate buffer 50 mM (pH 7), centrifuged, and resuspended in the same buffer to an optical density of approximately 0.7 (A_0_). In a glass tube, 1.5 mL of cellular suspension was mixed with 1.3 mL of hexadecane, vortex-mixed for 1 min, and left to rest for 9 min to obtain a separation of the two phases. The aqueous phase containing cells was transferred to a tube and the absorbance at 600 nm (A) was measured. The results are given as the percentage of adhesion: % adhesion = 1 − (A/A_0_).

The surface tension (mN·m^−1^) was quantified using cell-free supernatant with a Tensiometer K20 (KRÜSS GmbH, Hamburg, Germany) at room temperature, according to Du Noüy ring method (Gudiña et al. 2012). Before quantification, the equipment was calibrated with ultra-pure water, and all measurements were performed in triplicate.

Microbial lipids were quantified in lyophilized cells by the phospho-vanillin colorimetric method as described by Lopes et al. (2019). Results were expressed as microbial lipids content (mass of lipids per gram of cell dry weight, %) and microbial lipids concentration (by multiplying the lipids content by biomass concentration in the medium). The analysis of fatty acids composition was performed by gas chromatography after extraction of fatty acids from lyophilized cells with chloroform and further methylation using a mixture of methanol acidified with sulfuric acid (85:15, v/v), and pentadecanoic acid was used as the internal standard (Pereira et al. 2021). The relative amount of each fatty acid (%, w/w) was determined as the ratio between its concentration (g·L^−1^) and the sum of the concentrations of all fatty acids identified in the sample.

HC were extracted by liquid–liquid extraction using hexane as solvent (1:6, v/v) and undecane (C11) as the internal standard and quantified by gas chromatography using helium as the carrier gas at 1 mL·min^−1^. The temperature of the injector and FID detector were, respectively, 285 °C and 300 °C. The initial temperature of the oven was maintained at 60 °C for 1 min, followed by a gradual increase at a rate of 8 °C per minute until reaching 300 °C. The system was maintained at 300 °C for 5 min and then gradually decreased to 60 °C at a rate of 40 °C per minute (Miranda et al. 2024).

### Statistical analysis

All data represent the mean of two independent replicates. Statistical analysis was performed with Statgraphics Centurion XVI Version 16.2.04 (StatPoint Technologies Inc., USA), using one-way analysis of variance (ANOVA) and Tukey’s multi-range test to identify statistically significant differences in mean values (95 % level of confidence).

## Results

### Batch cultures

**Fig. 1:**
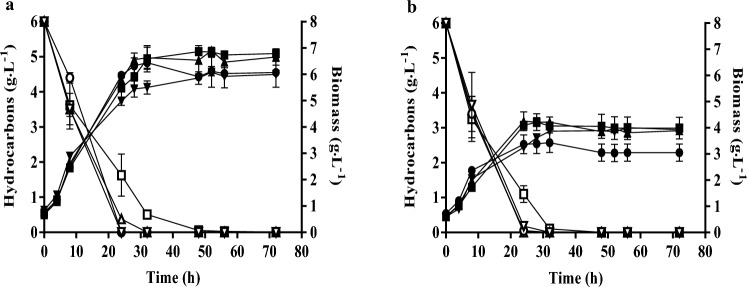
Biomass concentration (closed symbols) and total hydrocarbon consumption (open symbols) obtained in batch cultures of (**a)** *Y. lipolytica* CBS 2075 and (**b)** *Y. lipolytica* DSM 8218 with different controlled dissolved oxygen concentrations (% of saturation): 90 (▼,▽), 70 (●, ○), 50 (▲, ∆) and 30 (■, □). The error bars represent the standard deviation of two independent replicates

*Yarrowia lipolytica* CBS 2075 and *Y. lipolytica* DSM 8218 grew in a 6 g·L^−1^ HC mixture (2 g·L^−1^ hexadecane, 2 g·L^−1^ hexadecene, and 2 g·L^−1^ dodecane) without inhibition and no lag phase. Regardless of the yeast strain, DOC in the 30 % to 90% saturation had no significant effect on cell growth (Fig. 1) and biomass yield (Table 1). In all DOC conditions, a specific growth rate of approximately 0.16 h^−1^ and 0.14 h^−1^ was obtained in the exponential phase (first 8 h of growth) of CBS 2075 and DSM 8218. The final biomass concentration and biomass yield achieved in *Y. lipolytica* CBS 2075 cultures were, approximately, twofold higher than those obtained with the DSM 8218 strain.

**Table 1:** Biomass yield (*Y*_X/S_), the global uptake rate of hydrocarbons (*R*_HC_), maximum lipids concentration (Lipids_max_), lipids yield (*Y*_L/S_), volumetric lipid productivity (*Q*_L_), and specific rate of lipids synthesis (*q*_L_) obtained in *Y. lipolytica* CBS 2075 and *Y. lipolytica* DSM 8218 batch cultures with different controlled dissolved oxygen concentrations (DOC, % of saturation)

*Y. lipolytica* strain	DOC (%)	*Y*_X/S_ (g·g^−1^)	*R*_HC_ (g·L^−1^·h^−1^)	Lipids_max_ (g·L^−1^)	*Y*_L/S_ (g·g^−1^)	*Q*_L_ (g·L^−1^ h^−1^)	*q*_L_ (h^−1^)
CBS 2075	90	0.86 ± 0.09^a^	0.245 ± 0.002^ab^	0.8 ± 0.1^a^	0.13 ± 0.01^a^	0.033 ± 0.002^ab^	0.0066 ± 0.0003^a^
	70	0.89 ± 0.03^a^	0.254 ± 0.001^a^	0.74 ± 0.05^a^	0.13 ± 0.01^a^	0.031 ± 0.002^ab^	0.005 ± 0.001^a^
	50	1.00 ± 0.03^a^	0.23 ± 0.02^ab^	0.9 ± 0.2^a^	0.17 ± 0.03^a^	0.04 ± 0.01^b^	0.007 ± 0.001^a^
	30	1.01 ± 0.01^a^	0.17 ± 0.03^b^	0.9 ± 0.1^a^	0.154 ± 0.004^a^	0.019 ± 0.001^a^	0.0027 ± 0.0001^b^
DSM 8218	90	0.55 ± 0.02^a^	0.24 ± 0.01^a^	0.61 ± 0.02^a^	0.105 ± 0.002^a^	0.026 ± 0.002^a^	0.008 ± 0.001^a^
	70	0.47 ± 0.04^a^	0.242 ± 0.005^a^	0.5 ± 0.1^a^	0.05 ± 0.01^b^	0.011 ± 0.003^b^	0.003 ± 0.001^b^
	50	0.55 ± 0.01^a^	0.245 ± 0.002^a^	0.6 ± 0.1^a^	0.09 ± 0.01^a^	0.023 ± 0.003^a^	0.0055 ± 0.0002^c^
	30	0.6 ± 0.1^a^	0.19 ± 0.02^b^	0.50 ± 0.02^a^	0.101 ± 0.003^a^	0.021 ± 0.001^a^	0.0051 ± 0.0001^c^

Data are average ± standard deviation of two independent replicates. Values followed by the same letter in each column for each yeast strain do not present statistically significant differences (95 % confidence level)

*Y*_X/S_ was expressed as the mass of dry cells per mass of total hydrocarbons mixture consumed after 72 h; *Y*_L/S_ was expressed as maximum lipid concentration per mass of total hydrocarbons mixture consumed; *Q*_L_ was expressed as maximum lipid concentration per time; *q*_L_ was expressed as volumetric lipid productivity per mass of dry cells at the respective time

In this work, the global uptake rate of HC (*R*_HC_) by *Y. lipolytica* CBS 2075 was similar in the range of DOC tested (Table 1), and all HC were assimilated before the end of cultivation time (Fig. 1a). Likewise, *Y. lipolytica* DMS 8218 also assimilated all HC in the first 32 h (Fig. 1b). Dodecane, hexadecane, and hexadecene were assimilated at similar uptake rates in all DOC conditions in *Y. lipolytica* CBS 2075 cultures. However, dodecane assimilation by *Y. lipolytica* DSM 8218 in 30 % DOC cultures was, approximately, fivefold lower than the uptake rate of hexadecane and hexadecane.

**Fig. 2:**
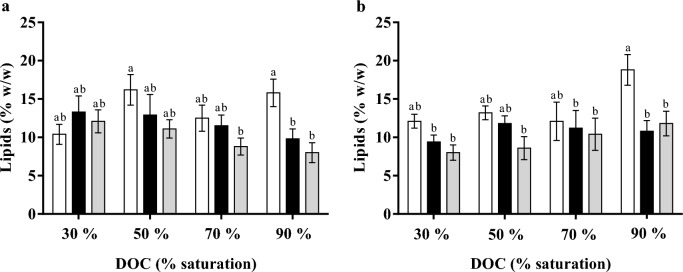
Lipids content (%) of (**a)** *Y. lipolytica* CBS 2075 and (**b)** *Y. lipolytica* DSM 8218 cells at 24 h (white bars), 48 h (black bars), and 72 h (grey bars) obtained in different conditions of controlled dissolved oxygen concentration (DOC, % saturation). The error bars represent the standard deviation of two independent replicates. Bars with the same letter do not present statistically significant differences (*p* ≥ 0.05)

Generally, in both *Y. lipolytica* cultures, the lipids content decreased from 24 h to 72 h. However, this decrease was only statistically significant at 90 % of DOC (Fig. 2). Due to the high biomass production in cultures of *Y. lipolytica* CBS 2075, the maximum lipids concentration (g·L^−1^) achieved with this strain was higher, regardless of the DOC studied. Specifically, at 30 % DOC, the concentration of the lipids was 44 % higher than that obtained in cultures of *Y. lipolytica* DSM 8218. Regardless of the yeast strain, DOC did not affect lipid yield (*Y*_L/S_) but higher *Y*_L/S_ were reached with the CBS 2075 strain. Particularly at 30 % DOC, the lipid yield in the CBS 2075 culture was 34 % higher than that obtained in the *Y. lipolytica* DSM 8218 culture. In general, the volumetric lipid productivity (*Q*_L_) was higher in CBS 2075 cultures and DOC had a distinct effect depending on yeast strains. The lowest value of *Q*_L_ was obtained at 30 % DOC in CBS 2075 cultures, whereas for DSM 8218 the minimum was attained at 70 % DOC. A 2.4-fold decrease in the specific rate of lipids synthesis (*q*_L_) was observed in CBS 2075 culture by decreasing DOC from 90 % to 30 %. In DSM 8218 cultures, the highest *q*_L_ was reached at 90 % DOC (Table 1).

Regardless of the DOC, higher proteolytic activities were obtained in *Y. lipolytica* CBS 2075 cultures compared to the DSM 8218 strain (Table 2). In CBS 2075 cultures, DOC had no significant effect on protease activity, reaching the maximum value at 28 h, which coincided with the stationary growth phase, remaining constant until the end. By contrast, an enhancement in protease was observed by increasing DOC from 30 % to 90 % in *Y. lipolytica* DSM 8218 cultures. However, the production profile was similar, and the maximum activity obtained at 24 h lasted constant until the end of cultivation.

In *Y. lipolytica* CBS 2075 cultures, an eightfold improvement in lipolytic activity was observed by increasing the DOC from 30 % to 90 %. In *Y. lipolytica* DSM 8218 cultures, lipase secretion was observed only at 90 % DOC (Table 2). Regardless of yeast strain and DOC conditions, the maximum activity was obtained at 8 h of cultivation, after which a decay in activity was observed.

### Pulse fed-batch cultures

#### HC consumption and production of lipids and protease

The results obtained in batch cultures demonstrated that both *Y. lipolytica* strains can assimilate 6 g·L^−1^ of HC mixture, directing them towards lipid-rich biomass and extracellular protease production. Moreover, the cell growth reached the stationary phase after 24 h (DSM 8218 strain) and 32 h (CBS 2075 strain), coinciding with the total assimilation of HC. To achieve high biomass concentration while avoiding potential inhibitory effects by increasing the initial HC concentration, several experiments were carried out with the addition of an HC mixture with one pulse at 48 h or with three pulses at 24 h, 48 h, and 72 h.

**Fig. 3:**
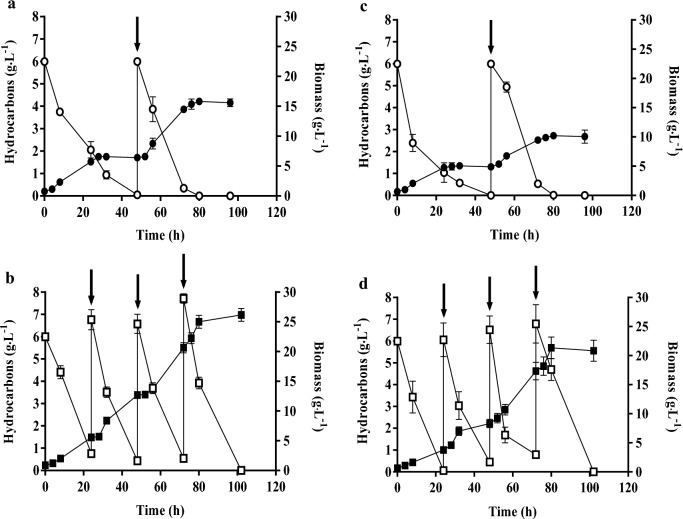
Biomass concentration (closed symbols) and total hydrocarbons consumption (open symbols) obtained in pulse fed-batch cultures of *Y. lipolytica* CBS 2075 (a, b) and *Y. lipolytica* DSM 8218 (c, d) with one pulse (●, ○) and three pulses (■, □). The error bars represent the standard deviation of two independent replicates. The arrows indicate the moment at which a pulse of concentrated HC-mixture was added to the culture

The maximum biomass concentration attained in CBS 2075 and DSM 8218 cultures after one pulse addition (Fig. 3a, c) was 57 % and 59 % higher, respectively than that obtained in batch cultures (Fig. 1). While a significant improvement (*p* < 0.05) in global biomass yield was observed in CBS 2075 cultures with the addition of one pulse, no significant effect was noted for the DSM 8218 strain when compared to batch cultures. Following the pulse addition, both yeast strains entered a second exponential growth phase only 4 h after the pulse. The addition of an HC pulse enhanced biomass production rate in CBS 2075 cultures, and an increase from (0.22 ± 0.01) g·L^−1^·h^−1^ (batch stage of 48 h) to (0.29 ± 0.01) g·L^−1^·h^−1^ was achieved after the pulse addition (Fig. 3a). By contrast, in DSM 8281 cultures, no significant differences were found in the biomass production rate during 1st batch stage (0.16 ± 0.03) g·L^−1^·h^−1^ and after the pulse addition (0.17 ± 0.02) g·L^−1^·h^−1^ (Fig. 3c).

**Table 2:** Maximum lipase (Lip_max_) and protease (Prot_max_) activities obtained in *Y. lipolytica* CBS 2075 and *Y. lipolytica* DSM 8218 batch and pulse fed-batch cultures

Mode of operation		*Y. lipolytica* CBS 2075		*Y. lipolytica* DSM 8218	
		Lip_max_ (U·L^−1^)	Prot_max_ (U·L^−1^)	Lip_max_ (U·L^−1^)	Prot_max_ (U·L^−1^)
Batch	90 % DOC	149 ± 2^a^	766 ± 74^a^	8.1 ± 0.1	374 ± 21^a^
	70 % DOC	81 ± 12^b^	554 ± 36^a^	n.d.	182 ± 33^ab^
	50 % DOC	22 ± 2^c^	566 ± 51^a^	n.d.	166 ± 22^ab^
	30 % DOC	16 ± 2^c^	538 ± 89^a^	n.d.	135 ± 20^b^
Pulse fed-batch	One pulse	n.d.	1583 ± 46^a^	n.d.	1353 ± 104^a^
	Three pulses	n.d.	3423 ± 70^b^	n.d.	3170 ± 137^b^

Data are average ± standard deviation for two independent replicates. Values followed by the same letter in each column for each mode of operation do not present statistically significant differences (95 % confidence level)

*n.d.* not detectable

After the pulse addition, all HC were completely assimilated by both *Y. lipolytica* strains in 32 h, coinciding with the beginning of a second stationary growth phase (Fig. 3a, c). Due to the high biomass concentration at the time of the pulse addition (48 h), faster assimilation of HC was observed compared to the 1st batch phase. For *Y. lipolytica* CBS 2075, the global uptake rate increased from (0.20 ± 0.02) g·L^−1^·h^−1^ to (0.224 ± 0.002) g·L^−1^·h^−1^ with the pulse addition (Fig. 3a). With DSM 8218, an increase from (0.19 ± 0.02) g·L^−1^·h^−1^ to (0.23 ± 0.02) g·L^−1^·h^−1^ was attained (Fig. 3c).

An improvement in maximum biomass concentration of 40 % and 52 % was obtained in experiments with three pulses with CBS 2075 and DSM 8218 strains, respectively, compared to experiments with only one pulse. Both yeast strains assimilated a total amount of 24 g·L^−1^ HC, in total of the 1st batch phase and three pulses. Moreover, the maximum biomass concentration of CBS 2075 and DSM 8218 cultures with three pulses (Fig. 3b, d) was, respectively, 74 % and 80 % higher than that attained in batch cultures (Fig. 1). It is noteworthy that pulse fed-batch cultures enable the maintenance of a high number of viable cells since cells of *Y. lipolytica* promptly entered a new exponential phase after each pulse (Fig. 3b, d), resulting in an increased biomass production rate. In CBS 2075 cultures, a significant increase in biomass production rate was achieved with the addition of pulses compared to the 1st batch phase (24 h). A biomass production rate of (0.23 ± 0.01) g·L^−1^·h^−1^ was attained in the first 24 h of cultivation, and after the 1st and 2nd pulses this value increased to (0.30 ± 0.01) g·L^−1^·h^−1^ and (0.33 ± 0.01) g·L^−1^·h^−1^, respectively. After the 3rd pulse, a remarkable biomass production rate of (0.55 ± 0.02) g·L^−1^·h^−1^ was attained since 4.7 g·L^−1^ of biomass was produced in only 8 h (from 72 h to 80 h of cultivation) (Fig. 3b). Regarding DSM 8218 cultures, a biomass production rate of (0.16 ± 0.01) g·L^−1^·h^−1^ was attained within 24 h (1st batch), and no significant differences were found in the biomass production rate (0.19 ± 0.02) g·L^−1^·h^−1^) reached after 1st pulse. However, biomass production rate increased after the 2nd (0.38 ± 0.03) g·L^−1^·h^−1^) and 3rd (0.50 ± 0.04) g·L^−1^·h^−1^) pulses, being 2.4-fold and 3.1-fold higher than that obtained in the 1st batch (Fig. 3d). The global biomass yield was similar in *Y. lipolytica* DSM 8218 cultures, regardless of the number of pulses, but a slight decrease in biomass yield was observed for CBS 275 strain by adding three pulses of HC (Table 3). As was observed in batch cultures, higher biomass concentration was reached in *Y. lipolytica* CBS 2075 cultures than in *Y. lipolytica* DSM 8218 cultures (Fig. 3).

**Table 3:** Global biomass yield (*Y*_X/S_), maximum lipids concentration (Lipids_max_), lipids yield (*Y*_L/S_), volumetric lipid productivity (*Q*_L_), and specific rate of lipids synthesis (*q*_L_) obtained in *Y. lipolytica* CBS 2075 and *Y. lipolytica* DSM 8218 pulse fed-batch cultures

*Y. lipolytica* strain	Strategy	*Y*_X/S_ (g·g^−1^)	Lipids_max_ (g·L^−1^)	*Y*_L/S_ (g·g^−1^)	*Q*_L_ (g·L^−1^·h^−1^)	*q*_L_ (h^−1^)
CBS 2075	One pulse	1.24 ± 0.03^a^	2.5 ± 0.1^a^	0.210 ± 0.002^a^	0.026 ± 0.001^a^	0.0017 ± 0.0001^a^
	Three pulses	1.05 ± 0.03^b^	4.3 ± 0.3^b^	0.18 ± 0.02^a^	0.045 ± 0.004^b^	0.0017 ± 0.0002^a^
DSM 8218	One pulse	0.78 ± 0.04^a^	0.9 ± 0.1^a^	0.07 ± 0.01^a^	0.009 ± 0.001^a^	0.0009 ± 0.0001^a^
	Three pulses	0.84 ± 0.04^a^	3.4 ± 0.2^b^	0.14 ± 0.01^b^	0.045 ± 0.003^b^	0.002 ± 0.001^b^

Data are average ± standard deviation for two independent replicates. Values followed by the same letter in each column for each strain do not present statistically significant differences (95 % confidence level)

*Y*_X/S_ was expressed as the mass of dry cells per mass of total hydrocarbons mixture consumed after 96 h; *Y*_L/S_ was expressed as maximum lipid concentration per mass of total hydrocarbons mixture consumed; *Q*_L_ was expressed as maximum lipid concentration per time; *q*_L_ was expressed as volumetric lipid productivity per mass of dry cells at the respective time

**Fig. 4:**
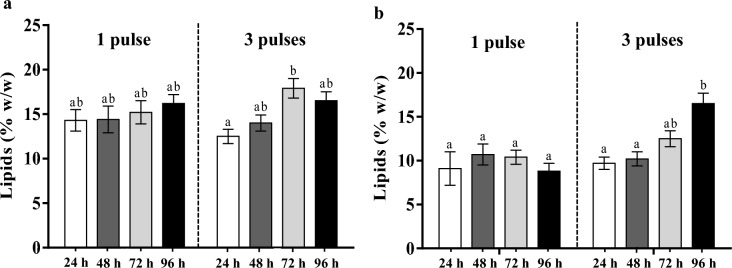
Lipids content (%) of (**a)** *Y. lipolytica* CBS 2075 and (**b)** *Y. lipolytica* DSM 8218 cells at 24 h, 48 h, 72 h, and 96 h obtained in pulse fed-batch cultures. The error bars represent the standard deviation of two independent replicates. Bars with the same letter for each yeast strain do not present statistically significant differences (*p* ≥ 0.05)

In experiments with a single pulse, adding an HC-mixture did not enhance the lipids content (%, w/w), which remained similar throughout the cultivation period in both CBS 2075 and DSM 8218 cultures (Fig. 4a, b). In contrast, in experiments with three pulses, the lipids content attained at 72 h (after two pulses addition) in CBS 2075 cultures was 1.4-fold higher than that obtained at 24 h (batch phase) (Fig. 4a). In the DSM 8218 culture, there was a 1.7-fold increase in lipids content at 96 h (after three pulses addition), compared to that achieved at 24 h (1st batch cycle) (Fig. 4b).

In pulse fed-batch experiments, a significant improvement in maximum lipids concentration was achieved (Table 3) compared to those obtained in batch cultures (Table 1), owing to the high biomass concentration reached. By contrast, *Y*_L/S_, *Q*_L,_ and *q*_L_ were overall similar to the values obtained in batch cultures, regardless of the *Y. lipolytica* strain.

In experiments with a single pulse, a three and twofold enhancement in maximum lipids concentration was obtained in cultures of CBS 2075 and DSM 8218, respectively, compared to those reached in batch cultures. Furthermore, three pulses led to a five and sevenfold improvement in maximum lipids concentration for CBS 2075 and DSM 8218 cultures, respectively, compared to cultures without pulses. The increase in the number of pulses from one to three led to a clear enhancement of the maximum lipid production by both strains (Table 3). The maximum lipids concentration attained in CBS 2075 and DSM 8218 cultures was, respectively, 42 % and 71 % higher than that reached with one pulse. The amount of lipids produced by *Y. lipolytica* DSM 8218 in the experiments with one pulse and three pulses were, respectively, 60 % and 21 % lower than those reached in cultures with CBS 2075 strain (Table 3). In CBS 2075 cultures, the lipid yield was statistically equal for both pulse strategies, whereas a twofold increase was reached in DSM 8218 cultures by increasing the number of pulses (Table 3). In addition, *Y*_L/S_ values reached with the CBS 2075 strain were higher than those obtained in cultures of *Y. lipolytica* DSM 8218. Particularly in experiments with one pulse, the lipid yield was 66 % higher than that obtained in *Y. lipolytica* DSM 8218 cultures. A two and fivefold increase in the volumetric lipid productivity was obtained in CBS 2075 and DSM 8218 cultures, respectively, by increasing the number of pulses. Whereas no significant differences were found in the specific rate of lipids synthesis for CBS 2075 cultures, a 2.2-fold increase was obtained in DSM 8218 cultures with three pulses (Table 3).

Lipids accumulated by *Y. lipolytica* CBS 2075 in both pulses’ strategies were predominantly composed of palmitic (C16:0) and oleic (C18:1) acids, followed by linoleic (C18:2) and palmitoleic (C16:1) acids, with low amounts of stearic (C18:0) and cis-10-heptadecenoic (C17:1) acids (around 5 % for both cases) (Table 4). In experiments with one pulse, strain DSM accumulated lipids rich in palmitic (C16:0), palmitoleic (C16:1), and linoleic (C18:2) acids, but lipids produced after the addition of three pulses were predominantly composed of palmitic (C16:0) and palmitoleic (C16:1) acids. Moreover, in cultures with three pulses, the content of C16:0 was 1.5-fold higher, while the content of C18:2 was 1.7-fold lower than those attained with one pulse (Table 4). Regardless of the pulse strategy, *Y. lipolytica* DSM 8218 accumulated around three times lower C18:1 than CBS 2075 strain. The unsaturated/saturated fractions of lipids produced by CBS 2075 and DSM 8218 strains were similar for the same pulse strategy studied, but lower unsaturated fractions were attained by increasing the number of pulses, regardless of the *Y. lipolytica* strain (Table 4).

**Table 4:** Fatty acids composition of microbial lipids produced by *Y. lipolytica* CBS 2075 and *Y. lipolytica* DSM 8218 in pulse fed-batch cultures carried out in an STR lab-scale bioreactor in conditions of 30 % DOC

*Y. lipolytica* strain	Strategy	Relative fatty acid content (%)							
		C16:0	C16:1	C17:1	C18:0	C18:1	C18:2	UFA	SFA
CBS 2075	One pulse	27 ± 1^a^	17 ± 1^a^	5.4 ± 0.5^a^	5 ± 1^a^	31 ± 1^a^	16 ± 1^a^	69 ± 2	31 ± 2
	Three pulses	31.5 ± 0.3^a^	14.8 ± 0.2^a^	4.8 ± 0.2^a^	5 ± 1^a^	31 ± 1^a^	13.3 ± 0.2^a^	63 ± 3	37 ± 2
DSM 8218	One pulse	26 ± 1^a^	30 ± 2ª	4.4 ± 0.2^a^	2.7 ± 0.4^a^	11 ± 1^a^	27 ± 3^a^	72 ± 1	28 ± 1
	Three pulses	38 ± 1^b^	28 ± 1ª	3.4 ± 0.1^b^	2.4 ± 0.2^a^	12.4 ± 0.3^a^	16 ± 1^b^	60 ± 3	40 ± 2

Data are average ± standard deviation for two independent replicates. Values followed by the same letter in each column for each strain do not present statistically significant differences (95 % confidence level)

*UFA*: total of unsaturated fatty acids; *SFA*: total of saturated fatty acids

The protease production significantly increased by changing the cultivation mode from batch to pulse fed-batch, particularly for the DSM 8218 strain (Table 2). Protease activities reached in cultures performed with one pulse and three pulses were, respectively, 3- and 6-fold (strain CBS 2075), and 10- and 23-fold (strain DMS 8218) higher than those attained in batch cultures. Moreover, a twofold improvement in maximum protease activity was attained for both *Y*. *lipolytica* strains by increasing the number of pulses from one to three (Table 2). It is worth noticing that in pulse fed-batch, protease production by the DSM 8218 strain was similar to that obtained for the CBS 2075 strain, contrary to what was observed in batch cultures (Table 2). Regardless of the yeast strain and pulse fed-batch strategy, protease production followed a similar pattern to batch cultures, being the maximum value obtained at the beginning of the stationary growth phase (growth-associated production). Furthermore, after each HC addition, protease production accompanied the new exponential growth phase and increased until it reached the stationary phase.

#### Cells hydrophobicity and surface tension of the medium

Table 4 shows the surface tension of cells-free supernatant and hydrophobicity of *Y. lipolytica* CBS 2075 and DSM 8218 cells at 0 h and 96 h. Regardless of pulse fed-batch strategy, the surface tension of the medium significantly decreased in CBS 2075 and DSM 8218 supernatants.

In the presence of HC, the hydrophobicity of CBS 2075 cells increased by approximately 12 % and 10 % in experiments with one and three pulses, respectively, after 96 h of cultivation. For strain DSM 8218, an increase of approximately 10 % was observed with one pulse and three pulses, compared to the hydrophobicity of yeast cells grown in glucose (Table 5).

**Table 5:** Yeast cells hydrophobicity (hydrophobicity, %) and surface tension of the cell-free medium (ST, mN·m^−1^) obtained in *Y. lipolytica* CBS 2075 and *Y. lipolytica* DSM 8218 pulse fed-batch cultures

*Y. lipolytica* strain	Strategy	Hydrophobicity (%)		ST (mN·m^−1^)	
		0 h	96 h	0 h	96 h
CBS 2075	One pulse	75 ± 1^a^	88 ± 1^b^	47.8 ± 0.4^a^	42.4 ± 0.2^b^
	Three pulses	74 ± 1^a^	84 ± 1^b^	47.3 ± 0.5^a^	37.2 ± 0.2^b^
DSM 8218	One pulse	69 ± 2^a^	79 ± 2^b^	47.5 ± 0.2^a^	42 ± 1^b^
	Three pulses	68 ± 2^a^	79 ± 2^b^	47.1 ± 0.6^a^	41.1 ± 0.3^b^

Data are average ± standard deviation for two independent replicates. Values followed by the same letter in each line for each parameter do not present statistically significant differences (95 % confidence level)

## Discussion

In this work, DOC had no significant effect on cell growth (Fig. 1) and biomass yield (Table 1) in cultures of *Y. lipolytica* CBS 2075 and *Y. lipolytica* DSM 8218. However, according to Fukuda (2023), the metabolic pathway of *n*-alkanes in *Y. lipolytica* involves a final step where fatty acids are activated to acyl-CoAs. Subsequently, these molecules undergo metabolism into acetyl-CoA through β-oxidation, a metabolic pathway that is highly reliant on a sufficient oxygen supply. Earlier studies showed that *Y. lipolytica* CBS 2075 assimilated 5 g·L^−1^ of hexadecane within 24 h at 600 rpm, enabling the maintenance of DOC nearly to 90 % of saturation (Miranda et al. 2024). It is noteworthy that the global uptake rate of HC obtained in this study surpasses those reported in the literature for bacterial species utilizing hexadecane at flask-scale (Tzintzun-Camacho et al. 2012; Zhong et al. 2014; Castro et al. 2017; Noveiri et al. 2023), which are the microbial species extensively studied for the biodegradation of HC.

In literature, the effect of DOC on lipids production by *Y. lipolytica* is far from consensual. Additionally, there are no studies in the literature reporting the impact of DOC on lipids production by *Y. lipolytica* strains from an HC mixture. Some authors recognize the significance of low oxygen availability for lipids synthesis by *Y. lipolytica* from both hydrophobic and hydrophilic substrates (Papanikolaou et al. 2002, 2007; Lopes et al. 2018, 2019; Pereira et al. 2021; Fabiszewska et al. 2022), while other researchers reported that a high lipid content is achieved in highly aerated cultures (Bellou et al. 2014; Fabiszewska et al. 2021). Under DOC of 90 %, the reduction in lipids content likely occurred due to intracellular lipids mobilization (lipids turnover) (Fig. 2), coinciding with the total depletion of the HC mixture observed at 24 h (Fig. 1). According to some authors, the mobilization of the intracellular lipids in *Y. lipolytica* strains appears to be associated with the complete depletion of the carbon sources from the medium (Dias et al. 2023). Furthermore, highly aerated cultures seem to enhance lipid mobilization inside the yeast cells (Magdouli et al. 2018).

Overall, the values of *Y*_L/S_, *Q*_L,_ and *q*_L_ were similar in batch and pulse fed-batch cultures, regardless of the *Y. lipolytica* strain (Tables 1, 3). The lipid yields obtained in this study are comparable to those found in the literature for *Y. lipolytica* KKP 379 and engineered *Y. lipolytica* AJD pAD-DGA1 growing in olive oil, glucose, and crude glycerol (Dobrowolski et al. 2019; Fabiszewska et al. 2021). Moreover, they are higher than those observed for *Y. lipolytica* cultures in glucose (Carsanba et al. 2020) and volatile fatty acids (Llamas et al. 2020). The *Q*_L_ values obtained herein were similar to those achieved by *Cryptococcus psychrotolerans* IITRFD growing in aromatic hydrocarbons (naphthalene, anthracene, and pyrene) (Deeba et al. 2018). Although in this study DOC had no significant effect on lipid yield, in fed-batch cultures of lipid-engineered *Y. lipolytica* JMY4086 with crude glycerol as substrate, higher values of *Y*_L/S_ and *Q*_L_ were reached under unregulated oxygen conditions, compared to experiments carried out at 50 % controlled DOC and highly aerated cultures (Rakicka et al. 2015). By contrast, in cultures supplemented with waste fish oil, a significant increase in the values of *Y*_L/S_ and *Q*_L_ was reached with controlled DOC (maintained above 20 % of saturation by varying agitation rate) since higher quantities of lipids were produced by *Y. lipolytica* KKP 379 (Fabiszewska et al. 2021). Regardless of the yeast strain, the specific rates of lipids synthesis (*q*_L_) obtained herein were similar or even higher compared with that reported by other *Y. lipolytica* strains growing in glucose/stearin mixture (Papanikolaou et al. 2006) and olive oil (Vasiliadou et al. 2018).

Lipids accumulation is intrinsically correlated with citric acid production in *Y. lipolytica* cultures since nitrogen-limited conditions (higher C/N ratio) are simultaneously required for lipids and citric acid synthesis. Given that, the synthesis of lipids can redirect metabolism away from citric acid production, and vice versa (Papanikolaou et al. 2020). By contrast, other authors found citric acid synthesis by *Y. lipolytica* independent of the C/N ratio (Ferreira et al. 2016; Cavallo et al. 2020). Although works were reporting the production of citric acid by *Y. lipolytica* from *n*-paraffin (Crolla and Kennedy 2004) and hexadecane (Finogenova et al. 2005), citric acid was not secreted in this study, suggesting that microbial metabolism was toward the accumulation of lipids alternatively to the synthesis of citric acid.

In CBS 2075 cultures, DOC had no significant effect on protease activity as already observed for this yeast strain when cultured in hexadecane as the sole carbon source (Miranda et al. 2024). In this context, it is noteworthy that low DOC values are suitable for achieving high proteolytic activities, which is significant for potentially lowering operational costs during the scale-up of the bioprocess, as it implies lower power input requirements. As observed in this study for *Y. lipolytica* DSM 8218, an increase in protease activity under high DOC was also attained in cultures of *Y. lipolytica* W29 growing in glucose (Lopes et al. 2009) and waste cooking oils (Lopes et al. 2019).

High oxygenation conditions have been shown to enhance lipase synthesis by *Y. lipolytica* growing in waste oils (Alonso et al. 2005; Snopek et al. 2021) and olive oil (Lopes et al. 2008). It seems that the activity of the *LIP*2 promoter, responsible for the secretion of the main extracellular lipase in *Y. lipolytica*, decreases under low DOC (Kar et al. 2010).

To enhance lipid-rich biomass and extracellular enzyme production in both *Y. lipolytica* strains, a pulse fed-batch strategy was studied at 30 % DOC, in which one or three pulses of concentrated HC medium were applied. It should be highlighted that both yeast strains consumed a total of 12 g·L^−1^ of HC in 80 h (Fig. 3), as a result of a single pulse addition at 48 h, which is a remarkable and uncommon feature in other microbial species (Zhong et al. 2014; Castro et al. 2017; SadrAzodi et al. 2019).

The results presented in this work suggest that three pulses of concentrated HC medium were an effective strategy to enhance the amount of lipids accumulated by *Y. lipolytica* strains. The maximum lipids concentration obtained herein for both *Y. lipolytica* strains, ranging from 1 g·L^−1^ to 4.3 g·L^−1^, are comparable or even higher than those found in the literature for *Y. lipolytica* growing in hexadecane (Miranda et al. 2024), pentadecane and heptadecane (Matatkova et al. 2017), volatile fatty acids (Pereira et al. 2021, 2022; Morales-Palomo et al. 2022), vegetable oils refinery wastewaters (Louhasakul and Cheirsilp 2013; Darvishi et al. 2019; Sarris et al. 2019), or synthetic medium mimicking lignocellulosic biomass hydrolysate (Dias et al. 2023).

In this study, the fatty acids composition of lipids was highly dependent on *Y. lipolytica* strain (Table 4). Lipids produced by *Y. lipolytica* CBS 2075 from hexadecane (Miranda et al. 2024) were richer in C16:0 and C16:1, but considerably lower amounts of C18:1 (17 %) and C18:2 (8 %) were attained when compared to those reached herein. According to Fickers et al. (2005), fatty acids synthesized after metabolization of some chain-numbered alkanes from 14 to 18 carbon atoms could be directly used for lipids synthesis and transported and stored in lipid bodies inside the yeast cells. This may explain the presence of an unusually high amount of palmitoleic acid (15 % – 30 %), since the HC used as a carbon source (hexadecane and hexadecene) may have been channeled directly to lipid bodies, resulting in lipids rich in fatty acids with a similar number of carbon atoms (16) as the HC assimilated by the yeasts. In *Y. lipolytica* CCY 30-26-36, the authors observed that a significant improvement in pentadecanoic acid (C15:0) was obtained in cultures using pentadecane as a carbon source, while in the presence of heptadecane, lipids produced had a high content of heptadecanoic acid (C17:0), probably due to metabolization of these alkanes into fatty acids precursors with unusual number of carbon atoms, such as propionyl-CoA (Matatkova et al. 2017).

Regardless of the pulse strategy, the lipids accumulated by *Y. lipolytica* CBS 2075 have a fatty acids composition (C16:0 and C18:1 > 30 %) similar to palm oil (C16:0–42 %; C18:0–4 %; C18:1–41 %; C18:2–10 %) (Giakoumis 2018). This particular fatty acid composition makes the lipids produced by *Y. lipolytica* CBS 2075 a good feedstock for biodiesel production and/or a promising substitute for palm oil (e.g., palm olein and palm stearin) employed in food products manufacturing (Ahmad et al. 2016), since dried and killed *Y. lipolytica* biomass cultivated in biofuel wastes have already been recognized as safe by the current food regulations (Jach and Malm 2022). On the other hand, lipids produced by the DSM 8218 strain had a low content in C18:1, which is an unusual outcome in *Y. lipolytica* cultures (Naveira-Pazos et al. 2023). Lipids produced by *Y. lipolytica* W29 from other hydrophobic substrates such as waste cooking oils (Lopes et al. 2019; Fabiszewska et al. 2022), pork lard (Lopes et al. 2018), and palm oil mill wastewater (Louhasakul et al. 2021) had a high content in C18:1 (26 % – 60%). *Y. lipolytica* DSM 8218 appears as a promising candidate to produce lipid-rich biomass with high content of palmitoleic acid (C16:1) (around 30 %), a valuable monounsaturated fatty acid rarely found in high amounts in microbial lipids and known for its broad applications in pharmaceutical and cosmetics industries (Kolouchová et al. 2015).

Pulse fed-batch was a suitable mode of operation for both lipids and protease production by these *Y. lipolytica* strains. The maximum protease activity reached in this work was similar to other values found in the literature for *Y. lipolytica* strains growing in castor oil (Gomes et al. 2013) and olive oil-based medium (Lopes et al. 2008), and even higher than those reported for *Y. lipolytica* W29 cultivated in waste cooking oils (Lopes et al. 2019) and pure glucose (Lopes et al. 2009). In hexadecane-based cultures, *Y. lipolytica* CBS 2075 achieved a maximum protease activity of approximately 500 U·L^−1^ (Miranda et al. 2024), suggesting that protease production in *Y. lipolytica* cultures from HC depends on the cultivation mode, HC concentration, and HC type. The substantial production of protease by *Y. lipolytica* utilizing an HC mixture represents a promising approach that aligns environmental sustainability with the production of high-value products. Proteases, being significant industrial biocatalysts, find diverse biotechnological applications, particularly in industries related to wastewater treatment (Naveed et al. 2021). The low values of lipolytic activity (< 40 U·L^−1^) reached may be due to the simultaneous secretion of high amounts of protease since these extracellular enzymes are responsible for the degradation of lipases (Najjar et al. 2011).

In the literature, it has been reported that cell-free samples of *Y. lipolytica*, after cultivation in heptadecane (Kim et al. 1999), used engine oil (Yalçın et al. 2018), and petroleum (Csutak et al. 2015) were able to emulsify different aliphatic and aromatic HC, as well as HC mixtures (e.g., crude oil), suggesting the secretion of biosurfactants by the yeast. A reduction in the surface tension of the medium significantly was observed in CBS 2075 and DSM 8218 supernatants (Table 5), possibly due to the production of biosurfactants by the yeasts, albeit in small amounts. Similar results of surface tension reduction (7 mN·m^−1^– 10 mN·m^−1^) were observed for *Y. lipolytica* IMUFRJ 50862 cultivated in crude oil (Ferreira et al. 2023). By contrast, Fontes et al. (2010) reported a lower reduction in the surface tension of the medium in the presence of hexadecane compared to glucose and glycerol. The authors suggested that *Y. lipolytica* IMUFRJ 50862 produced cell-bound biosurfactants rather than extracellular biosurfactants, as they observed the migration of *Y. lipolytica* cells from the aqueous phase (cultivation medium) to the organic phase (hexadecane).

Microbial adhesion to HC (MATH) is a parameter that provides insights into the alterations in microbial surface properties. As described by Rosenberg et al. (1980), microbial cell hydrophobicity is determined by the ability of cells to adhere to a hydrophobic surface, such as HC. Cells of *Y. lipolytica* CBS 2075 and DSM 8218 exhibited a high cell surface hydrophobicity after their growth in glucose (pre-inoculum), as occurred with other strains such as *Y. lipolytica* PG-20 and PG-32 in hydrocarbons (Hassanshahian et al. 2012), and *Y. lipolytica* NCIM 3589 in glucose and bromobenzene (Vatsal et al. 2017). In addition, Chrzanowski et al. (2008) emphasize the crucial role of the hydrophobic character of *Y. lipolytica* cells during yeast cultivation in HC. Their observations highlight that *Y. lipolytica* strains displaying high cell surface hydrophobicity (> 76 %) have enhanced ability in assimilating HC such as dodecane and hexadecane.

## Conclusion

In summary, this study illustrates that *Y. lipolytica* can simultaneously assimilate higher alkanes (hexadecane and dodecane) and alkenes (hexadecene) while synthesizing valuable compounds. This represents an alternative approach for producing lipids-rich biomass and protease from pollutant compounds. In batch cultures, 30 % dissolved oxygen concentration was sufficient to achieve high yields of lipids and protease. Regardless of the *Y. lipolytica* strain, pulse fed-batch cultures improved the bioprocess performance. The addition of pulses of aliphatic hydrocarbons led to notable enhancements in biomass, lipids, and protease production compared to batch cultures. Lipids produced by *Y. lipolytica* CBS 2075 in pulse fed-batch cultures were rich in C16:0 and C18:1. Conversely, lipids from the DSM 8218 strain had a high content of C16:0, C16:1, and C18:1 with one pulse, while C16:0 and C16:1 were the main fatty acids with three pulses. Lipids produced by *Y. lipolytica* strains are a promising raw material for several industries, including food manufacturing, cosmetics, pharmaceuticals, and biodiesel. Regardless of the culture strategy studied, the presence of aliphatic hydrocarbons increased the cell surface hydrophobicity of *Y. lipolytica*. Furthermore, a reduction in the surface tension of the medium was observed, suggesting the production of extracellular biosurfactants, even at low amounts.

## Data Availability

No datasets were generated or analysed during the current study.

## Declarations

### Research involving human and/or animal participants

This article does not contain any studies with human participants or animals performed by any of the authors.
